# Electrochemical and statistical study of Nickel ion assessment in daily children intake samples relying on magnesium aluminate spinel nanoparticles

**DOI:** 10.1038/s41598-024-64052-1

**Published:** 2024-07-16

**Authors:** Maysa R. Mostafa, Gehad G. Mohamed, Omar A. Fouad

**Affiliations:** 1 Chemistry Department, Faculty of Science, Cairo University, Giza, 12613 Egypt; 2 Nanoscience Department, Basic and Applied Sciences Institute, Egypt-Japan University of Science and Technology, New Borg El Arab, Alexandria, 21934 Egypt

**Keywords:** Carbon paste electrode, Ceramic nanoparticles, Candy, Coca, Chocolate, Cocaine, Fizzy drinks, Scanning electron microscope, X-ray, Potentiometry, Nanoscale materials, Environmental chemistry

## Abstract

Lately, children's daily consumption of some products, such as cereals and candies, has been rising, which provides a compelling rationale for determining any metallic substances that may be present. Monitoring the concentration of certain metals, like nickel, in these products is necessary due to medical issues in humans when consumed regularly. So, in this work, a novel and highly selective carbon paste as a Ni(II) ion-selective sensor was prepared and investigated using ceramic magnesium aluminum spinel nanoparticles as the ionophore and tritolyl phosphate (TOCP) as a plasticizer. A modified co-precipitation method was used to synthesize the spinel nanoparticles. X-ray diffraction, scanning electron microscope with EDAX, transmission electron microscope, and BET surface area were used to determine the phase composition, microstructure, pores size, particle size, and surface area of the synthesized nanoparticles. The spinel nanoparticle was found to have a nano crystallite size with a cubic crystal system, a particle size ranging from 17.2 to 51.52 nm, mesoporous nature (average pore size = 8.72 nm), and a large surface area (61.75 m^2^/g). The composition ratio of graphite carbon as a base: TOCP as binder: spinal as ionophore was 67.3:30.0:2.7 (wt%) based on potentiometric detections over concentrations from 5.0 × 10^−8^ to 1.0 × 10^−2^ mol L^−1^ with LOD of 5.0 × 10^−8^ mol L^−1^. A measurement of 29.22 ± 0.12 mV decade^−1^ over pH 2.0–7.0 was made for the Nernstian slope. This sensor demonstrated good repeatability over nine weeks and a rapid response of 8 s. A good selectivity was shown for Ni(II) ions across many interferents, tri-, di-, and monovalent cations. The Ni(II) content in spiked real samples, including cocaine, sweets, coca, chocolate, carbonated drinks, cereals, and packages, were measured. The results obtained indicated no significant difference between the proposed potentiometric method and the officially reported ICP method according to the F- and t-test data. In addition to utilizing ANOVA statistical analysis, validation procedures have been implemented, and the results exceed the ICP-MS methodology.

## Introduction

Globally, metal pollution has garnered significant attention with the industry's rapid advancement. Since heavy metals are very soluble, the body may effectively store them, and most of them are known to be very harmful^1^. Industrial agriculture is one potential source of heavy metals in products; industrial farm runoff can accumulate in soil or water and then be absorbed by cocoa beans through their root system when growing^2,3^. Growing crops in the soil near volcanoes is also, consider as another potential source^2–4^.

Nickel is the fifth most common element on earth and is regarded as a fundamental minor component for humans^5^. There are several oxidation states for nickel, but the (2+) oxidation state is the one that biosystems use the most^6^. Additionally, plants and some domestic animals are thought to require nickel, the metal component of the urease enzyme. Stainless steels and other metal alloys, batteries, ceramics, electroplating, pigments, and catalysts are just a few of the products that employ nickel. Furthermore, nickel ion contamination may be present in chocolate and other semi-finished compounds, including cocoa of liquor, butter, and powder, which can withstand the post-harvest process^4^.

Organization of World Health (WHO) and the United States Environmental Protection Agency (USEPA) have established the maximum allowable concentration of nickel in drinking water at 0.07 and 0.04 mg L^-1^, respectively^5,7^. Nickel can cause medical issues such as asthma, irritation, cardiac and kidney infections, and pneumonic fibrosis degeneration. Workers heavily in contact with nickel have the highest risk of developing lung and nose malignancies. As a result, precisely determining nickel ions in bio-and environmental specimens is crucial to examining contamination of the environment and problems with public health^6^. Also, recently, the daily intake by children of cereals, candy, fizzy drinks, chocolate, and coca have increased, so it gives a powerful reason for the determination of metals such as nickel ions, which might be found in these products.

It is known that cocaine is an illegal substance, but it also has a lot of medical uses as an anesthetic^8^ but under medical control (healthy limited value). Due to the significant number of surprising products that contain cocaine, it might be used by children. So, it became urgent to determine Ni(II) ions in these products. Cocaine is found in nasal congestion^9^ and eye drops^10^, toothpaste^11^, soda products^11^ and others. All contain a small amount of cocaine^11^. Given the broad distribution of nickel toxicity in the ecosystem, there is an urgent need to develop innovative techniques for determining trace amounts of Ni(II) ions due to their influence on the health of humans.

Several approaches have been implemented to monitor the Ni(II) ions, including atomic absorption spectrometry, inductively coupled plasma with either mass spectrometry or optical emission spectrometry, and ultraviolet–visible methods^12–14^. However, significant restrictions exist, such as the necessity for training and the usage of advanced tools, as well as lower sensitivity. Electrochemical techniques are widely used because of their apparatus availability, procedural ease, rapidity, precision, and accuracy, making them ideal for determining Ni(II) ions. Potentiometric measurements using the ion-selective technique provide several advantages, including quick response, easy setup, a significant linear dynamical range, simplified processes, and low resistance. Several electrodes (ISEs) designed mainly for alkaline earth/alkali, heavy, and transition metal ions are now accessible for sale thanks to substantial research in this field.

With the generic formulas MAl_2_O_4_ (M = Mg, Zn, etc.) and MFe_2_O_4_ (M = Co, Mg, Mn, Ni, etc.), respectively, numerous nanoparticles of oxide with the XY_2_O_4_ [X is + 2 oxidation state cation, Y is + 3 oxidation state cation and O anion] structure crystallize as spinel aluminates and ferrites. Eight X, sixteen Y, and thirty-two oxygen atoms constitute a standard spinel structure^15^. In this close packing, 32 octahedral interstices and 64 tetrahedral interstices are surrounded by six O^2−^ ions and four O^2−^ ions, respectively. Typically, only 24 cations (8 X^2+^ and 16 Y^3+^) in standard spinel are occupied in the 96 interstices (64 tetrahedral and 32 octahedral) between the anions^16,17^. Spinel nanoparticles are superior to other materials for electrochemical sensors due to their conduction sites for electron transfer, high surface-to-volume ratio, mesoporous structure, and nano size. All the previous facts and findings encourage synthesizing the carbon paste ion selective electrode based on spinel nanoparticles. Many spinel oxides have been synthesized by different methods and used as a sensor like Ni–Zn ferrite spinel^18^, Li_1.05_Al_0.02_Mn_1.98_O_4_^19^, CoMn_2_O_4_^20^, spinel manganese(IV) oxide^21^ and CuCo_2_O_4_^22^. Among the spinel, magnesium aluminate spinel (MgAl_2_O_4_) has been the focus of researchers due to its properties such as high hardness (16 GPa), high mechanical strength both at room (135–216 MPa) as well as at elevated temperatures (120–205 MPa at 1300 °C), high resistance against chemical attack, high melting point (2135 °C), comprehensive band gap energy, high electrical resistivity, relatively low thermal expansion coefficient (9 × 10^−6^ °C^−1^ between 30 and 1400 °C), low dielectric constant (*ε* = 7.5) and high thermal shock resistance^16,23^. Magnesium aluminate (MgAl_2_O_4_) is found in different applications such as neutron radiation resistance, optically transparent ceramics, catalyst, refractory and catalyst support, etc^24^.

So, this work aims to verify a technique for simultaneously determining the concentration of heavy metals such as Ni(II) ion. Therefore, the main goal is to describe the synthesis and characterization of nano-magnesium aluminum spinel and characterize it by a variety of characterization techniques to shed light on the structure and behavior of the spinel, including powder X-ray diffraction (XRD), transmission electron microscope (TEM), scanning electron microscope (SEM), and Brunauer–Emmett–Teller (BET) analysis. The prepared spinel nanoparticles were used as a modifier in electrodes based on carbon paste to estimate Ni(II) ions in the numbers of actual specimens. The electrodes worked well as sensors for detecting Ni(II) in pure form and numerous actual samples. Spinel-NP amount, binder, H^+^ ion concentration, temperature, selectivity coefficient, and lifetime effects were thoroughly investigated. The parameters for method validation were examined. The methodology was validated using the following primary method validation criteria: limit of detection (LOD), limit of quantification (LOQ), linearity, and uncertainty measurement. The verified approach was then applied to cocoa, cereal, candy, and fizzy drink samples from smallholders and businesses. The ANOVA statistical analysis program was applied to validate the proposed potentiometric methos.

## Results and discussion

### XRD for the prepared nano- spinel (MgAl_2_O_4_)

**Figure 1:**
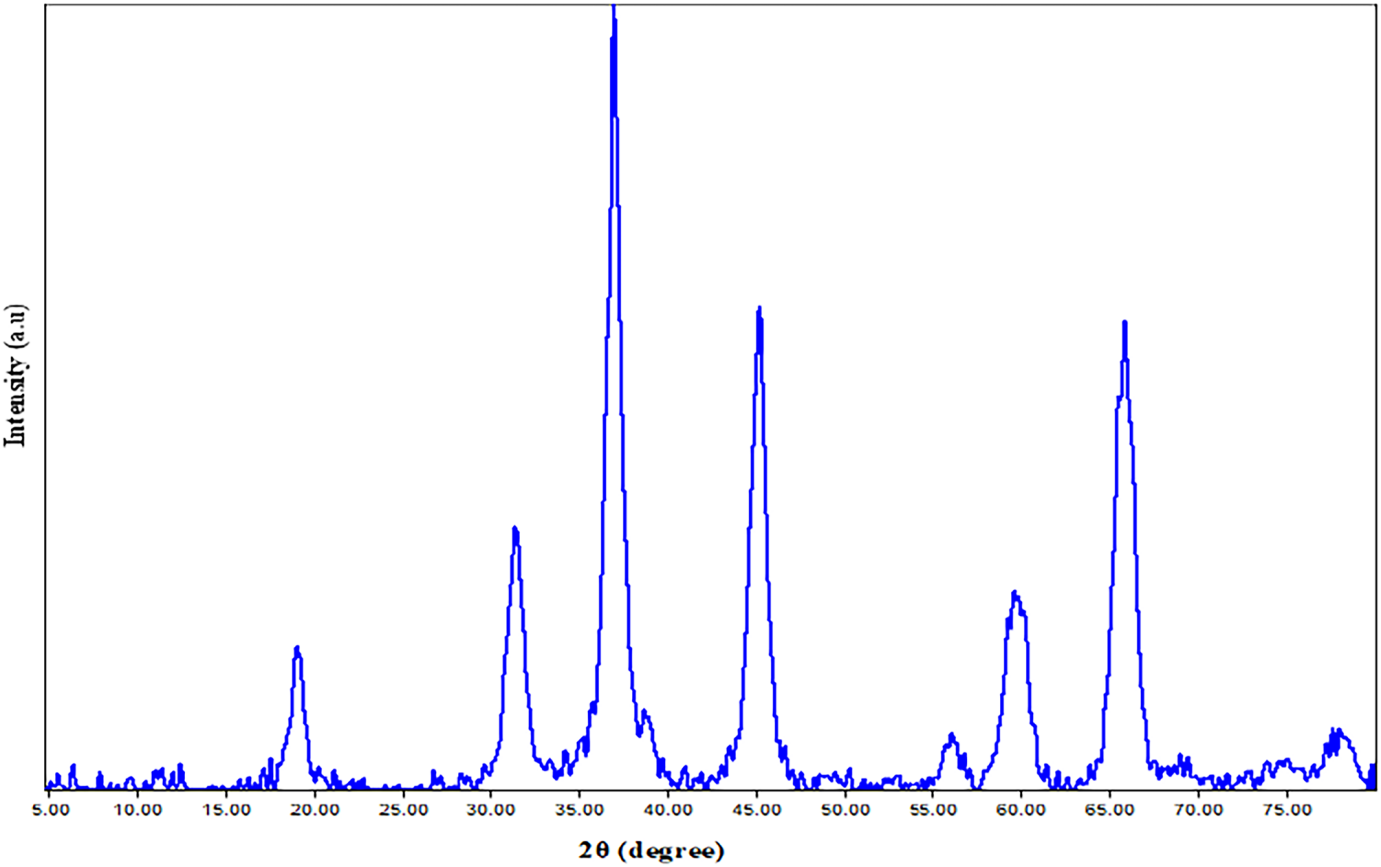
XRD patterns of the nano spinel (MgAl_2_O_4_).

The XRD pattern of nano magnesium aluminum spinel (MgAl_2_O_4_) is displayed in Fig. 1. The spinel's cubic crystal structure is verified by the sharp diffraction peaks at two thetas: 18.9°, 31.3°, 36.8°, 44.7°, 55.6°, 59.3°, and 65.1°, which are ascribed to the (111), (220), (311), (400), (422), (511), and (440) planes, in that order. The card number is COD 9,002,850, and the space group is F d -3 m (227). Without any diffraction peaks linked to other phases, the predominant phase found is spinel, indicating the purity of the magnesium aluminum spinel (MgAl_2_O_4_). Furthermore, the Debye–Scherrer equation revealed that the typical crystallite size was in the 35 nm range^25,26^.

### The scanning electron microscope attached to the EDAX unit

**Figure 2:**
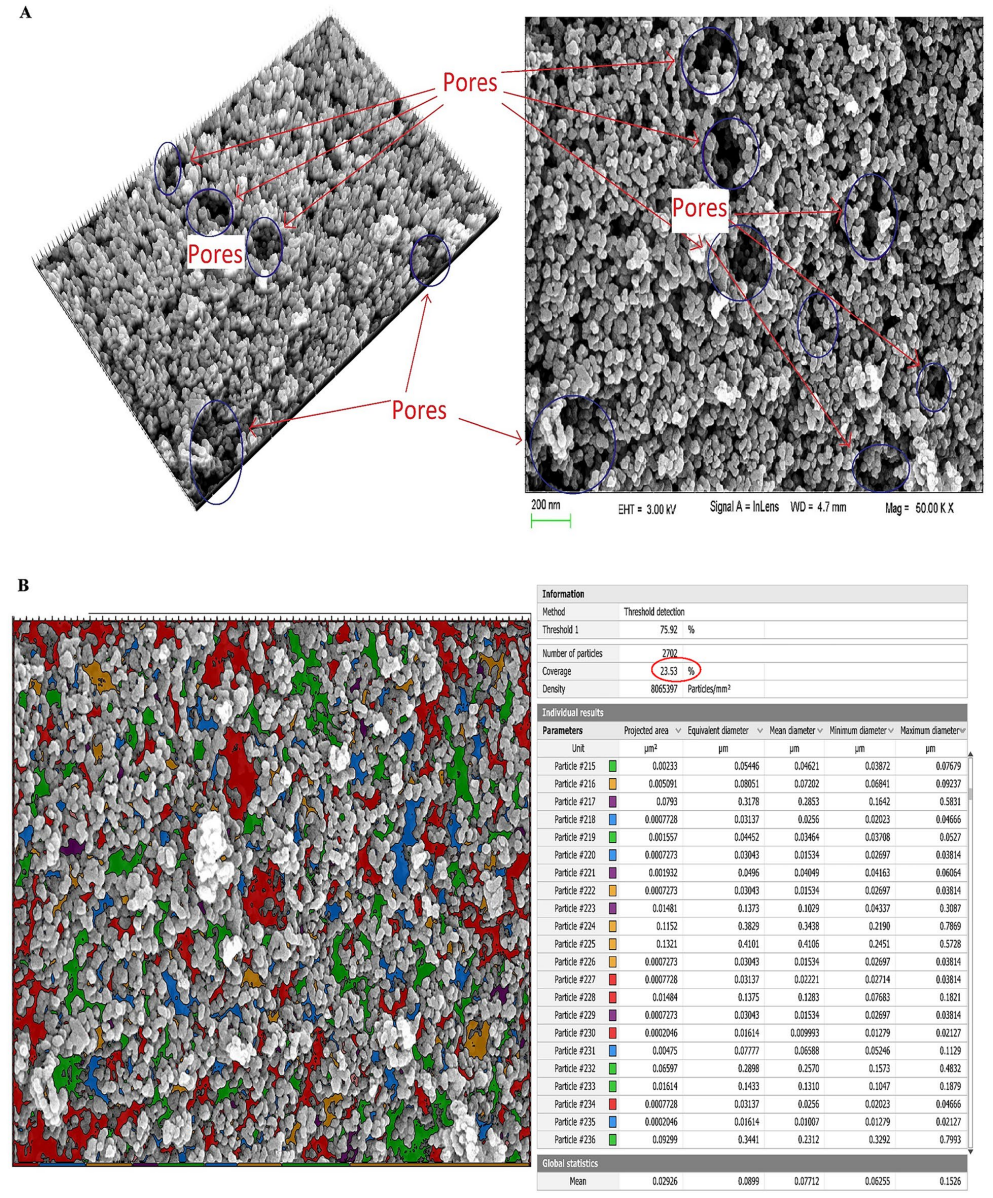

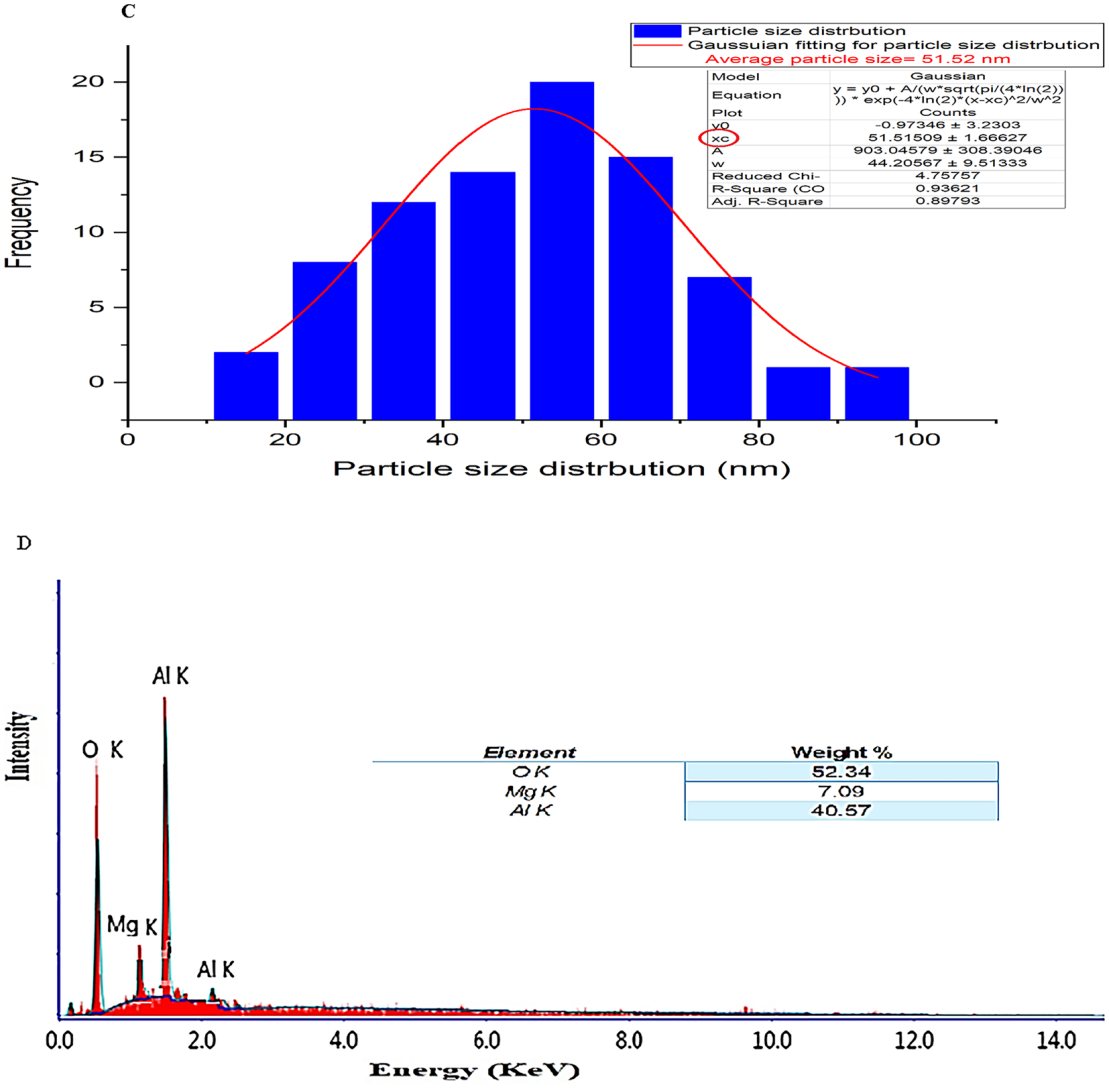
The SEM microphotographs (**A**), particle and pore size distributions (**B**, **C**), and EDAX data (**D**) of the prepared spinel nanoparticles.

Using a scanning electron microscope (SEM) in aggregation with EDAX, Digital Surf's Mountains Lab®, Java 1.8.0 172 with ImageJ (1.53e) software, the morphology and microstructure of the fabricated spinel nanoparticles were analyzed^27,28^. The spinel nanoparticles' smooth surface, porous construction, consistent matrix, and spherical-like form were all visible in the SEM images (Fig. 2A). The synthesized MgAl_2_O_4_ nanoparticles had a variety of pore diameters and particles, as seen by the SEM microphotographs.

As shown in Fig. 2B, the Digital Surf's Mountains Lab® software applied a color threshold method on 500 nm-scale SEM microphotographs, which allowed the pores in the material to take distinct colors and showed that the overall percentage of pores was about 23.53% according to the overall measured areas in 500 nm-scale SEM microphotographs. To estimate the distribution of particle sizes, the Gaussian mixture model and histogram in Fig. 2C were also generated using Java 1.8.0 172 with ImageJ (1.53e) tool. Spinel nanoparticles were discovered to have an average particle size of 51.52 nm. EDAX data was utilized to determine the chemical composition of spinel nanoparticles (Fig. 2D)^29^. The composition is primarily magnesium (7.09%), aluminum (40.57%), and oxygen (52.34%). In order to ascertain the uniformity of the distribution of the constituent elements (Al, Mg, and O) in the MgAl_2_O_4_ sample, the atomic percentages of all three elements were compared at various points. The EDAX results confirmed that the distribution of these elements is consistent throughout the sample.

### Transmission electron microscope (TEM) study

**Figure 3:**
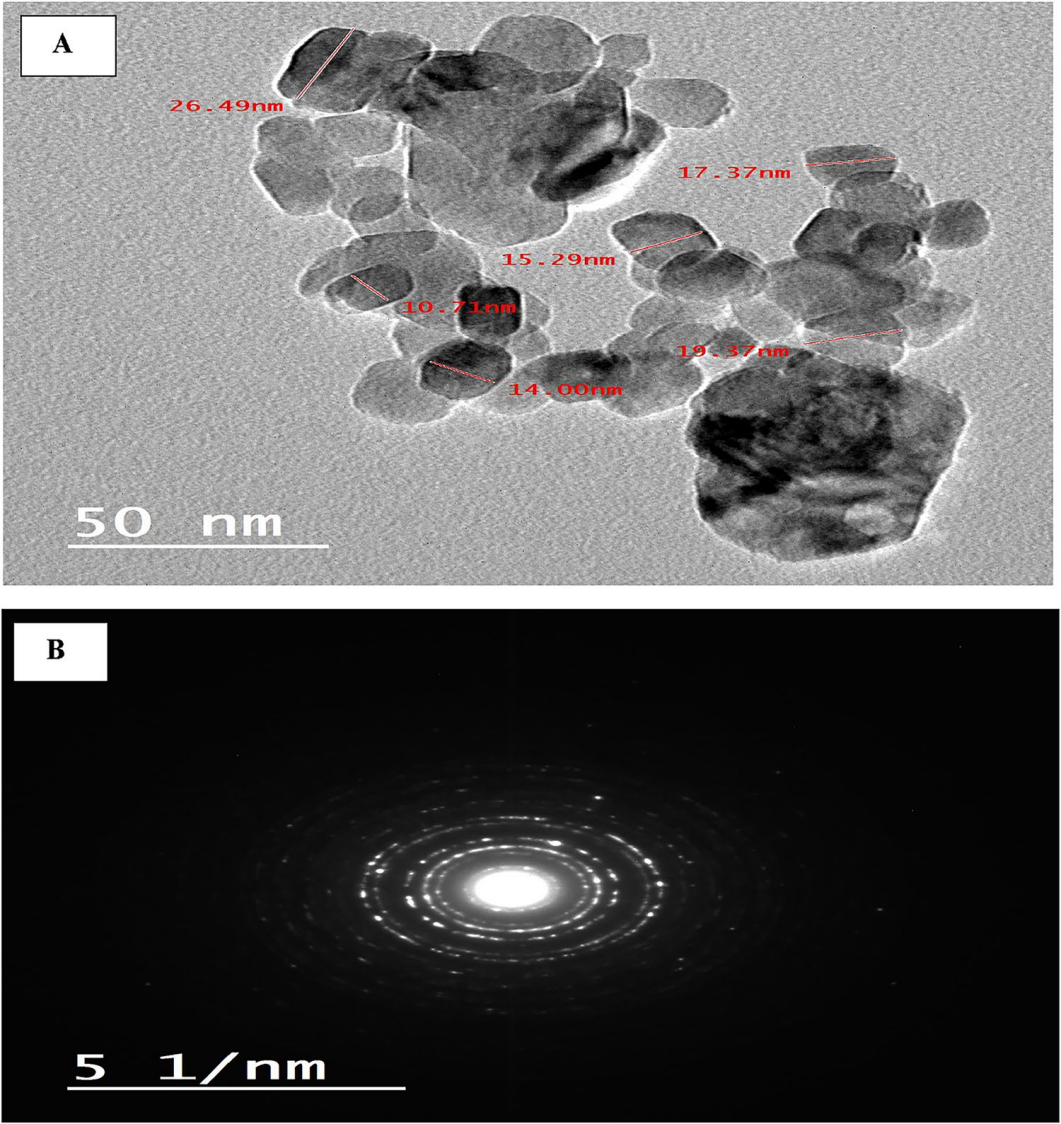
The synthesized spinel nanoparticles' high-resolution TEM image (HR-TEM) (**A**) and selected area electron diffraction (SAED) (**B**).

TEM image of the nano spinel (MgAl_2_O_4_) was displayed in Fig. 3 (A). The crystallite extent established from XRD data (mean crystallite extent = 35 nm) and SEM image evaluation (mean particle extent = 51.52 nm) are in reasonable conformity with the TEM image, which exposed a particle with a cubic constitute and a size range of 10.7 to 26.49 nm and a mean particle size of 17.2 nm. High levels of crystallinity in the synthesized nano spinel were also confirmed by the selected area electron diffraction (SAED) pattern Fig. 3 (B)^30^.

### Brunauer–Emmett–Teller (BET) analysis

The spinel nanoparticle (MgAl_2_O_4_) surface area, pore volume, and average pore size must be understood entirely regarding nitrogen adsorption–desorption. According to the IUPAC-1985 class of physisorption isotherms, the synthetic nano-spinel revealed a type IV-(a)-H3 hysteresis loop isotherm^31^. The N_2_ adsorption–desorption measurement (Supplementary Fig. S1) of the BET analysis indicated a significant rise in the adsorbed volume starting at P/P0 = 0.86. The surface area, mean pore diameter, and pore volume of the nano-spinel were 61.75 m^2^/g, 8.72 nm, and 0.256 cm^3^/g, respectively, according to the BET analysis, the spinel nanoparticle had a well-developed mesoporous feature with a high surface area^25,32, 33^.

After analyzing the synthesized nano spinel's characteristics, which pointed out its high surface area, nanoscale, and mesoporous composition, it can be exploited as an ionophore in ion-selective CPE to find the Ni(II) ion in actual samples.

### Impact of sensor composition

To acquire the electrochemical attitude, calibration took place by dipping the electrodes in combination with the dual junction Ag/AgCl reference electrode in solutions of Ni(II) ions that vary from 1.0 × 10^–9^ to 1.0 × 10^–2^ mol L^-1^. They were permitted to equilibrate by stirring before recording their e.m.f. measurements. The sensors demonstrated a straight-line response across the range of 5.0 × 10^–8^–1.0 × 10^–2^ mol L^-1^, which is a broader range than 1.0 × 10^–6^-1.0 × 10^–1^ mol L^-1^, as found in previously reported research^34^. Many modified CPEs were prepared using different contents of 5, 10, 15, and 20 mg of spinel nanoparticles with many plasticizers, namely DEHP, TOCP, DBP, o-NPOE, and DIBP. It was evident from Table 1 that CPEs modified by 10 mg of spinel nanoparticles and plasticized with TOCP gave a good Nernstian slope of 29.22 ± 0.12 mV decade^-1^.

**Table 1:** The performance of carbon paste electrodes with different spinel content.

No	Graphite (mg)	Spinel (mg)	Name of plasticizer	Plasticizer (g)	Slope (mV decade^-1^)	R^2^	Linear range (mol. L^-1^)
1	250	5	TOCP	0.1167	17.36 ± 1.21	0.809	1.0 × 10^−**4**^–1.0 × 10^−2^
2	250	10	TOCP	0.1167	29.22 ± 0.12	0.998	**5**.0 × 10^−8^–1.0 × 10^−2^
3	250	15	TOCP	0.1167	19.30 ± 1.11	0.911	1.0 × 10^−**4**^–1.0 × 10^−2^
4	250	20	TOCP	0.1167	18.79 ± 1.21	0.900	1.0 × 10^−5^–1.0 × 10^−2^
5	250	10	DIBP	0.0970	20.70 ± 0.33	0.817	1.0 × 10^−**5**^–1.0 × 10^−**2**^
6	250	10	DEHP	0.1040	26.50 ± 0.21	0.780	1.0 × 10^−**4**^–1.0 × 10^−**2**^
7	250	10	o-NOPE	0.1044	19.40 ± 1.20	0.939	1.0 × 10^−6^–1.0 × 10^−**3**^
8	250	10	DBP	0.1050	38.45 ± 0.59	0.966	1.0 × 10^−**4**^–1.0 × 10^−**2**^
9	250	0	TOCP	0.1167	15.07 ± 0.11	0.830	1.0 × 10^−6^–1.0 × 10^−**2**^

### Influence of solvent mediators

**Figure 4:**
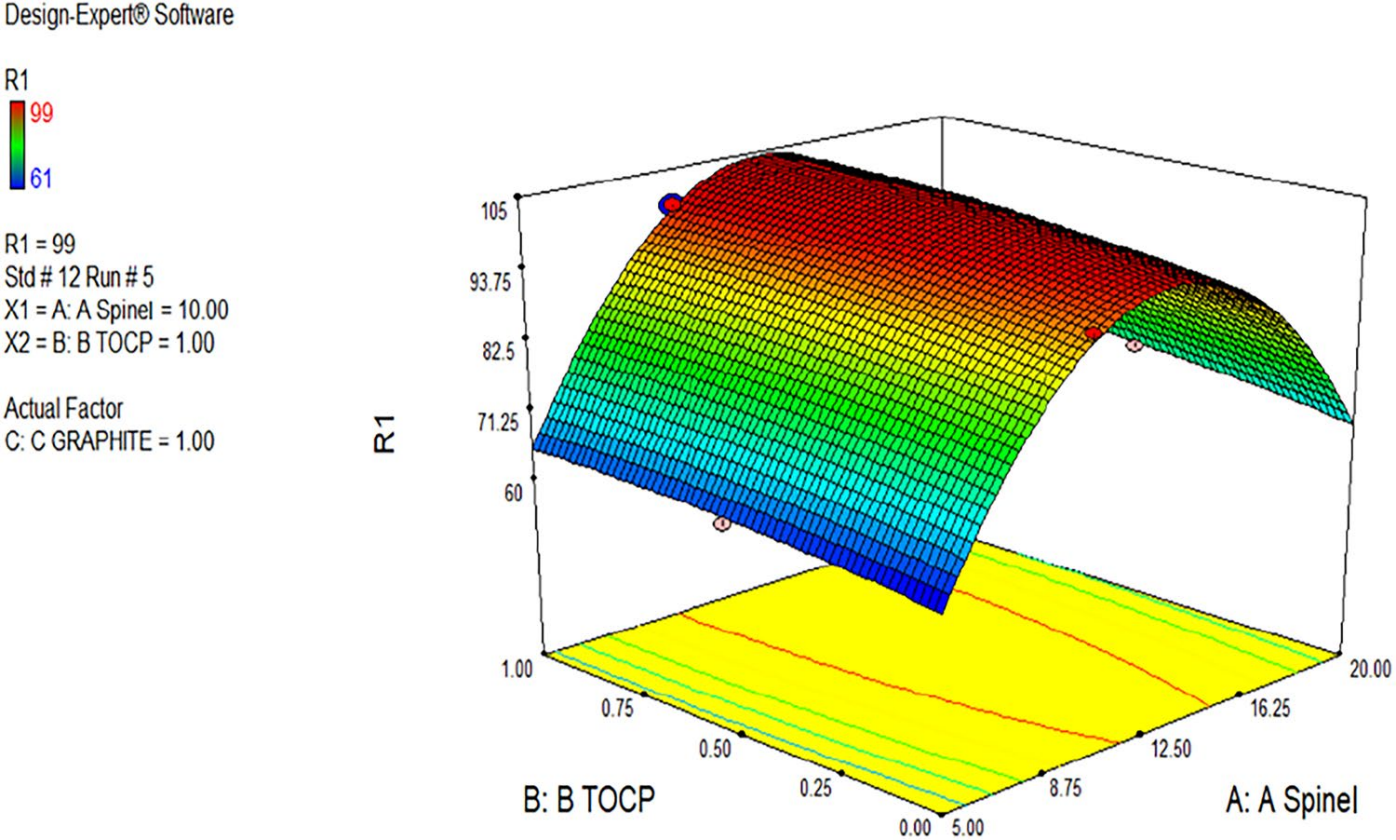
3D curve of the relation between ionophore (**A**, spinel), plasticizer (**B**, TOCP), and the response of the sensor (R1) with prediction equal 100.035%, which achieved the highest slope according to ANOVA prediction.

Solvent mediators (plasticizers) are thought to have a crucial influence on how it behaves of carbon paste (CP). They offer several benefits, including improved ionic mobility, increased sensing material solubility, and decreased total bulk resistance of the sensor due to the polarity properties. They enhanced the mechanical link between every one of the electroactive carbon particles into a homogenous compact combination^35,36^. Various solvent mediators (DEHP, TOCP, DBP, o-NPOE, DIBP) were subjected to potentiometric measurement, and the obtained findings are shown in Fig. 4. The optimal solvent mediator was chosen based on the modified CPE's potentiometric performance, which was determined using the Nernstian response and straight domains. The results showed that TOCP was the best solvent mediator for potentiometric measurement of Ni(II) ions. Table 1 showed that the electrode plasticized with TOCP only (electrode 9) cannot give a good response or an excellent Nernstian slope where it provided low slope. So, TOCP doesn't have a reason for the good response, as shown in electrode 2, and it can't be used to determine the Ni(II) ion. But electrode 2, which contained TOCP with spinel, was the best as it gave the highest Nernstian slope (29.22 ± 0.12 mV decade^-1^) than the others.

### Qualitative and quantitative computed optimization

**Figure 5:**
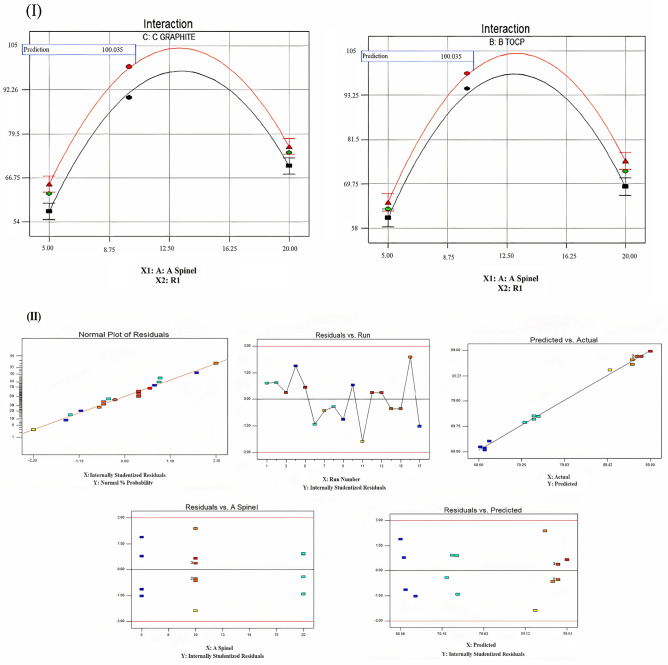
(I) The interaction between different factors, such as spinel (**A**), TOCP (**B**), and graphite (**C**) and (II), which is a normal distribution and actual versus expected values of experimental design using ANOVA prediction.

An original design aimed to determine the type of plasticizer (binder) and ionophore content (mg) best suited to the sensor performance. The design contained nine sensors and five distinct plasticizers (DEHP, TOCP, DBP, o-NPOE, and DIBP). The Design Expert® created a prediction model based on the experimental results. The ANOVA statistical evaluation model demonstrated that the kind of binder and amount of ionophore used in the paste substantially affect the obtained slopes (Table 2). The predicted slopes significantly correlate with the actual ones, as shown in Figs. 4 and 5, when TOCP and graphite are utilized in constant amounts. The highest slopes were produced when the percentage of tested ionophore was changed, progressing the model's maximum attractiveness. There is no interaction between any of these factors, even binder, ionophore, or graphite, as the low values of AB and AC proved which are 1.90 and 1.29, respectively, as shown in Fig. 5I, Table 2 and Fig. 5II which explained the relations between the predicted values against the actual values, normal probability of the values, and the residual values against different amounts of spinel ionophore. All figures' red squared shapes refer to values that achieve the highest desirability with a maximum ANOVA prediction of 100.035%. The ionophore was the most influential factor in the slope of the sensor due to its high value, which is listed in Table 2, which equals 231. All these proofs supported the ANOVA computed design.

**Table 2:**
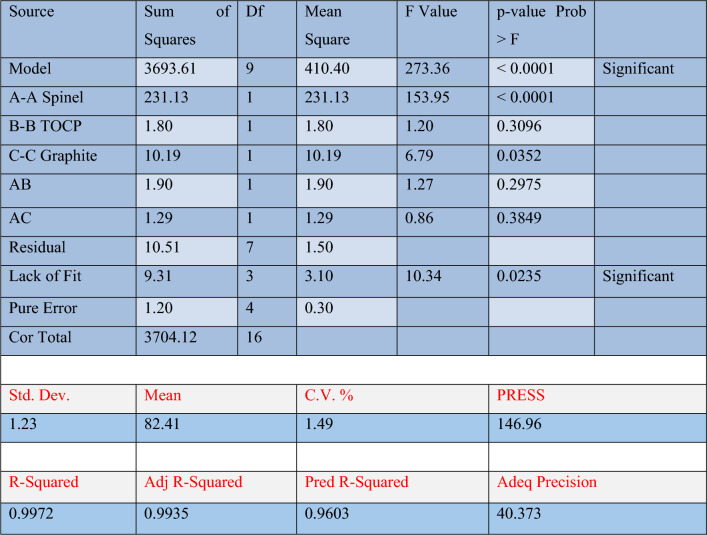
One-way ANOVA statistical evaluation of the slopes obtained from the sensors comprising the quantitative design.

The model F-value of 273.36 indicated that the model's prediction is significant. Values of "Prob > F" less than 0.0500 suggested that the model terms were essential. Values larger than 0.1000 denoted that the model terms were not significant. The "Lack of Fit F-value" of 10.34 indicated a considerable lack of fit. There is only a 2.35% possibility that a critical "Lack of Fit F-value" might be caused by noise. The "Pred R-squared" of 0.9603 is reasonably consistent with the "Adj R-squared" of 0.9935. "Adeq Precision" assesses the signal-to-noise ratio. A ratio larger than four is preferred. The ratio of 40.373 showed a valid signal. This model can help to explore the design space.

### Interaction between nanoparticles paste and Ni(II) ions

**Figure 6:**
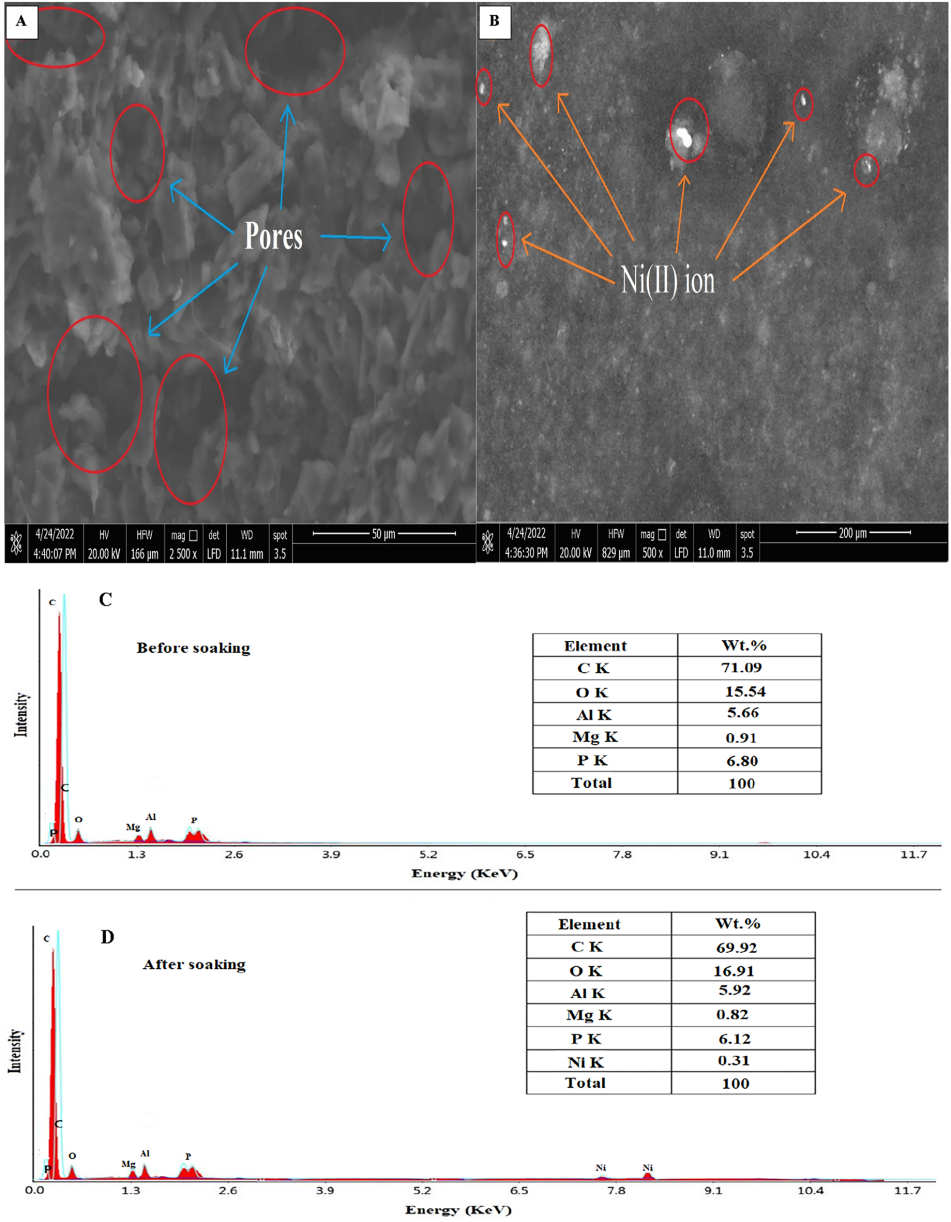
SEM and EDAX of the nanoparticles paste of the proposed electrode before (**A**, **C**) and after soaking (**B**, **D**).

Ceramic spinel nanoparticles are ionophore in carbon paste electrodes because they can attach Ni(II) ions in authentic samples or pure solutions. This connection will alter the contour of the electrode surface, making it rough and causing a pattern of white patches due to the presence of Ni(II) ions on the surface of the electrode upon soaking, as seen in the SEM picture (Fig. 6A,B). Also, the EDAX analysis agreed with this process, which was provided by the existence of Ni(II) ions in it, as well as quantitative information regarding surface composition before and after soaking in Ni(II) ion solution, as shown in (Fig. 6C,D).

### Effect of variation of H ^+^ concentration

**Figure 7:**
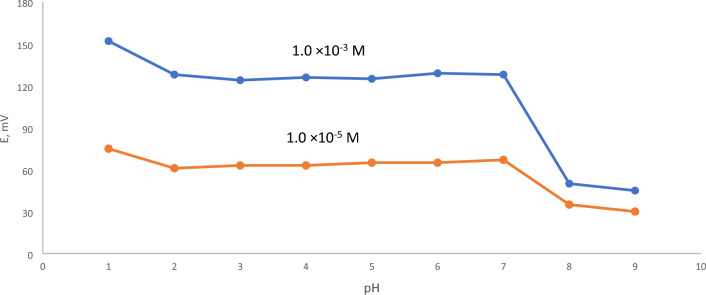
The potential response of the electrode at various pH values.

The influence of hydrogen ion concentration on the reaction behavior of modified Ni(II)-CPE is a significant aspect in ensuring the electrode's high performance. The potentials have been determined for 1.0 × 10^−3^ and 1.0 × 10^−5^ mol L^-1^ of Ni(II) ions at various pH levels (1–9). As previously stated in the experimental section, diluted solutions of HCl or NaOH were employed to modify the pH from 1 to 9. The findings revealed that the potential response remained consistent across the range of 2.0–7.0, which was adjusted using buffer solution as acetate or phosphate, as seen in Fig. 7. The improved Ni(II)-CPE functioned well in the pH range of 2.0–7.0, with minimal interference from H^+^ or, OH^−^ which is a broader pH range than shown in other previous studies that were 5–8^37^ or 6.5–8.5^38^. The shifts occurred at pH levels less than two or greater than 7, indicating decreased Ni(II)-CPE performance. This behavior could be clarified as an outcome of competition with hydrogen ions in high acidity and the formation of both soluble and insoluble Ni(II) ion hydroxyl complexes, which precipitated at high pH levels.

### Temperature study

The improved Ni-ISE's performance in test solutions was assessed at various temperatures (10, 25, 30, 40, 50, and 60 °C). The standard cell potentials (E°cell) were determined at different temperatures using the point intercepts of the relevant calibration plots at pNi(II) = 0. They were used to estimate the isothermal temperature coefficient (dE°/dt): *E*_cell_° = *E*_cell_° (25) + ( d*E*°/d*T*) (t–25).

The sensor gave a good Nernstian response within the temperature range of 10–60 °C. The straight–line slope was obtained from the relation of E_cell_° (y-axis) and t–25 (x-axis), where the isothermal coefficient of MCPE equals 0.957 mV/C. The small values of the isothermal coefficient showed high thermal stability. The standard potential of the reference electrode (Ag/AgCl) was calculated at the different temperatures using the following equation: y = 0.9486x + 197.85, *E*_cell_ = 197.85+ 0.9486 (t–25).

### Response time and lifetime study

**Figure 8:**
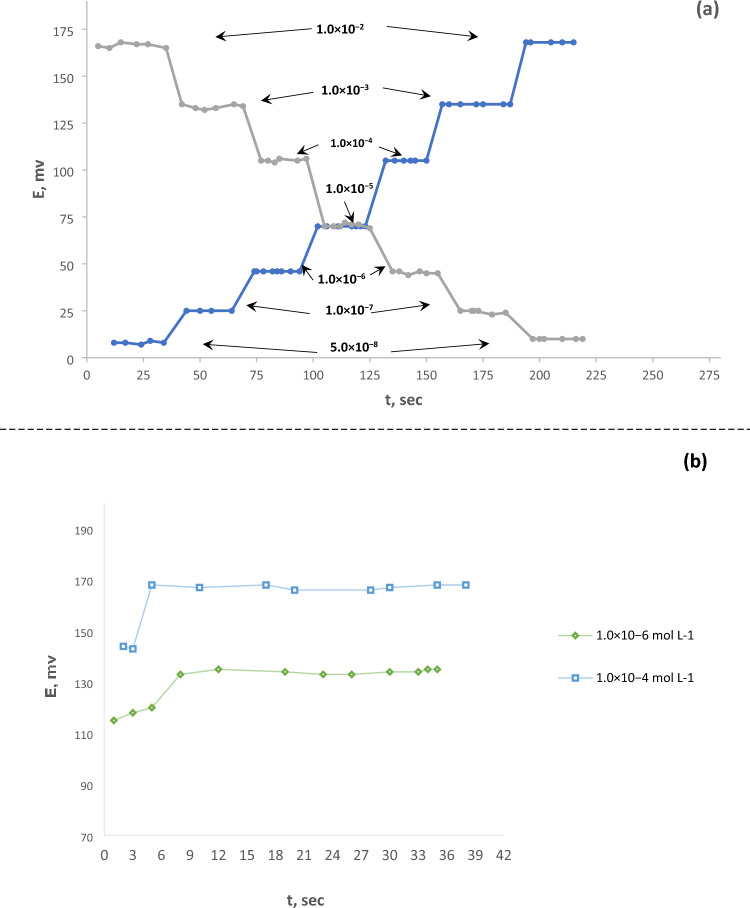
The response time of the optimal modified CPE. (**a**) represents the response time of the proposed electrode at different concentrations of Ni(II); at (**b**), two concentrations were chosen, and the duration of constancy was focused on them to explain the time needed to give a constant response.

**Figure 9:**
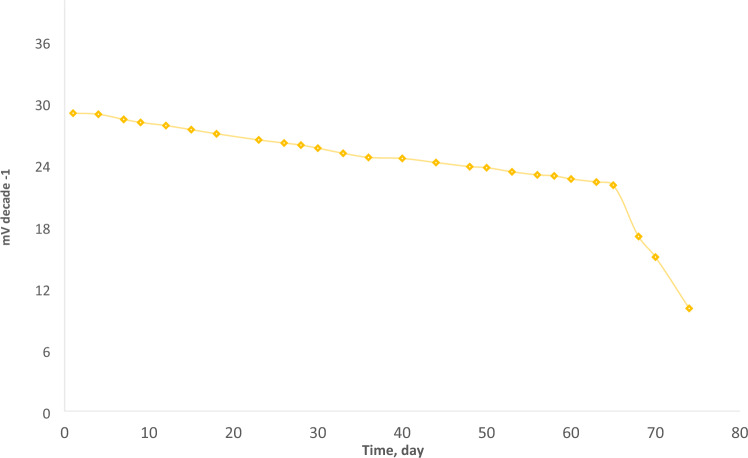
The lifetime of the paste containing spinel nanoparticles.

Response time and electrode life are critical components of any potentiometric sensor's analytical performance^39^. The average reaction time is the time it takes for a sensor to attain a stable voltage (± 1 mV) of the equilibrium value. This value was measured by dipping the electrode progressively in a series of target ion solutions. The average time for the electrode to achieve a constant potential response within ± 1 mV of the ultimate equilibrium value. The reaction time of the ideal modified CPE was measured by increasing the Ni(II) ion concentration from 5.0 × 10^−8^ to 1.0 × 10^−2^ mol L^−1^ (Fig. 8). As a result, the electrode reached the equilibrium value very quickly after ~ 8 s, which reflected the incorporation of the best content of spinel and good solvent mediator where it is equal to 11 s as found in the previous study^40^ or equal 10 s as in Alizadeh et al*.* report^34^. The lifetime was estimated by developing the calibration diagram under optimum conditions at an extended time, and it reached nine weeks without a critical change in the slopes of the calibration diagram, as shown in Fig. 9, that is a longer lifetime (3–5 weeks) as previously reported^37^.

### Selectivity coefficient study

The potentiometric selectivity factors were also calculated, which are the most essential factor in determining its characteristics. These coefficients have demonstrated that the electrode response varies in the presence of an interrupting ion (B) relative to the target Ni(II) ion. The selectivity coefficients were obtained using multiple approaches, including the matched potential method (MPM) and the separation solution method (SSM)^41–43^.

In SSM, the assessment is made by measuring the potential of the interfering and target ions in a cell of a sensor and a reference electrode. The alteration is made using two solutions: one of the desired ions (Ni(II)) in the absence of competing species (no B) and the E of it. The second solution includes the interfering species B (without the desired ion); hence E is obtained. For these cations, the selectivity parameters were obtained using the Eisenman technique^44^.

**Figure 10:**
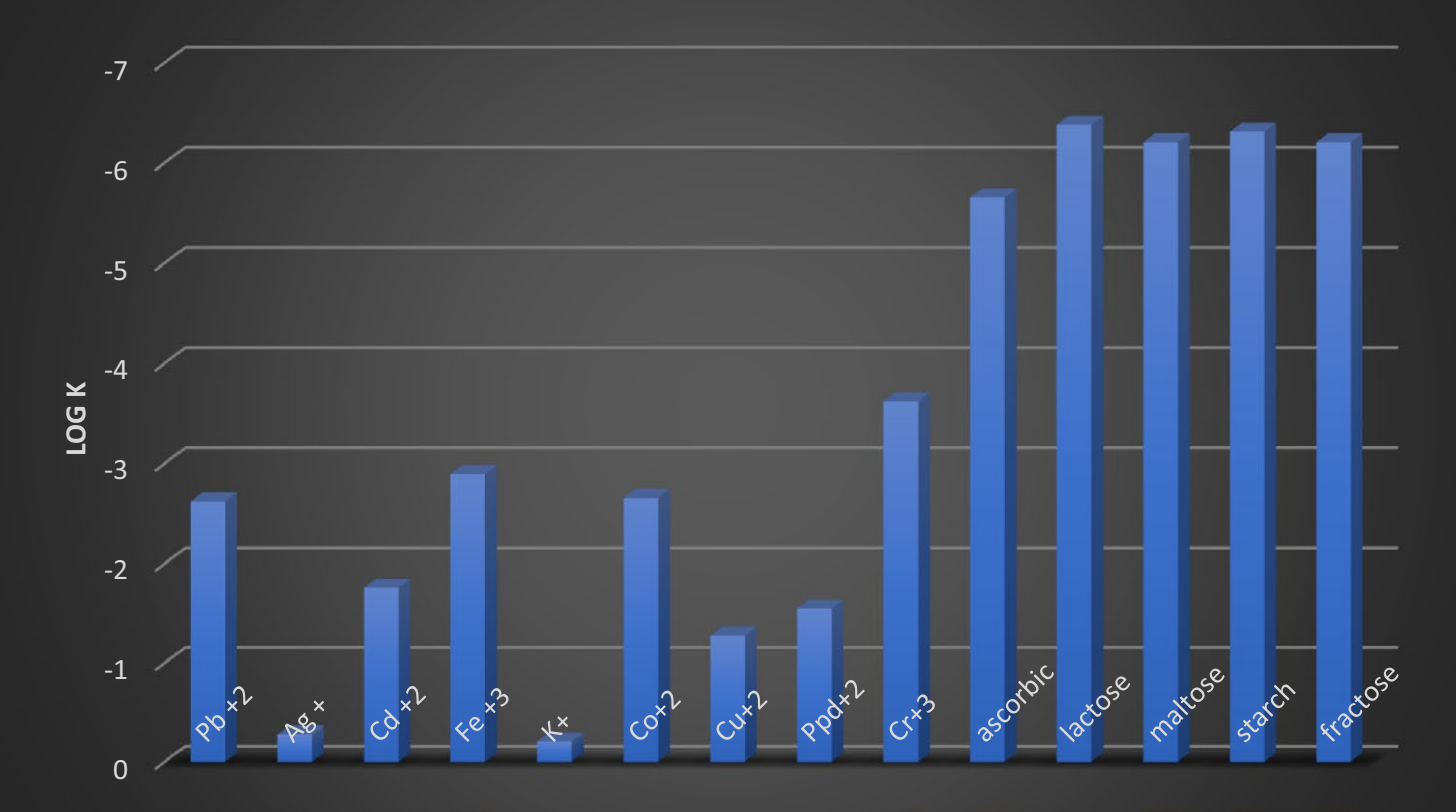
Selectivity coefficients for proposed electrode towards various inorganic cations, carbohydrates, and sugar**.**

In MPM, they define this as "the ratio of the main concentration of ions to the interrupting ion concentration that resulted in the same potential change as the reference solution." It does not obey the Eisenman equation. So, it is appropriate for neutral sugars and carbs such as maltose and starch^36^. Figure 10 showed that the produced electrode showed different selectivity for the target ion compared to the evaluated cations (alkali and transition metal ions). The selectivity coefficients (logK Ni(II)) for modified CPE towards various inorganic cations, carbohydrates, and sugars were tested using several techniques such as SSM "separated solution method" and "MPM "matching potential method." The results showed none of the evaluated interfering species could have a high selectivity coefficient.

This suggested that these ions have no significant impact on the reaction obtained with nickel ions. As a result, the improved sensor demonstrated high effectiveness and selectivity in detecting Ni(II) ions dissolved in diverse interfering materials. This may be explained in terms of ionic size variations, which affect mobilities and permeability. Sugars' remarkable selectivity is primarily due to the difference in polarity and lipophilic nature of their molecules compared with Ni(II), which causes no appreciable interference.

### Applications

#### Assessment of Ni(II) ions in authentic samples

The suggested sensor was examined to evaluate Ni(II) ions in various actual samples, such as cocaine, candy, coca, chocolate, fizzy drinks, cereals, and packages. Table 3 illustrated the recovery values of 97.07–101.30% with SD = 0.14–0.99 for the proposed sensor. Furthermore, the relative standard deviation (RSD%) values for all five replicate estimates were less than 5. These results supported the recommended sensor's repeatability, accuracy, and ability to recover quickly.

**Table 3:** Repeatability of the nickel(II) determinations using modified CPE.

Samples		Intra-day			Inter-day		
	Taken*	Found*	Recovery (%)	RSD (%)	Found*	Recovery (%)	RSD (%)
Ni(II)	27.30	27.02	98.97	1.19	27.16	99.49	0.52
Coca		26.96	98.75	1.64	26.76	98.02	1.87
Chocolate candy		26.56	97.29	3.12	26.68	97.73	3.71
		27.38	100.30	3.40	27.34	100.10	3.16
Fizzy drink		27.66	101.30	2.70	27.10	99.27	1.60
Cereals		27.5	100.70	1.59	27.00	98.90	2.42
Cocaine		26.86	98.39	2.33	26.50	97.07	2.88
Package		26.64	97.58	2.10	26.50	97.07	2.10

*μg mL^-1^.

The results obtained from the potentiometric calibration method were compared to those from the inductively coupled plasma mass spectrometry (ICP-MS) technique. This comparison obtained was summarized in Table 4, which showed recoveries values in the range from 96.00 to 99.85% and from 97.14 to 101.69% with SD = 0.05–1.00 and 0.04–0.09 for potentiometric calibration and ICP methods, respectively. Statistical evaluation of the results of the Ni(II) ion analysis in the pure solution and the spikes of some actual samples showed good recovery and relative standard deviation results, indicating the proposed electrode's high accuracy and precision. These results were also compared with the results of the ICP technique, which showed this method's advantages and no significant difference between the two techniques.

**Table 4:** Recovery and RSD of ICP against the Ni(II)-CPE.

Method	Sample	Taken	Found	Recovery	RSD%
		μg mL^-1^	μg mL^-1^		
Potentiometry	Chocolate	11.80	11.78	99.85	0.50
	Cereals	7.00	6.72	96.00	1.24
	Candy	5.20	5.10	98.08	1.96
ICP-MS	Chocolate	11.80	12.00	101.69	0.76
	Cereals	7.00	6.80	97.14	0.73
	Candy	5.20	5.09	97.88	0.10

At n = 5, 95% confidence level, F-test tabulated = 5.050, t-test tabulated = 2.571.

F-test experimental = 0.39–1.27, t-test experimental = 0.48–1.78.

### Method validation

Accreditation of electroanalytical procedures is a strategy created to determine if a determination methodology used for particular measurements is acceptable for its intended purpose and comparable to other techniques^45–47^. Using standard solutions, validation tests were conducted to validate accuracy, detection limits, measurement limits, linearity, precision, and specificity.

Accuracy and precision may be described as the values closest to the same when validated, regardless of whether they are traditional, accurate, or acceptable reference values and the value discovered. Precisions were measured intra-day and inter-day using four separate samples of two concentrations each. The relative standard deviations were found to be relatively minimal, indicating that the selected approach is reasonably repeatable, as shown in Table 3. As a result, the accuracy of the chosen potentiometric approach was calculated as a percentage relative standard deviation (RSD%) utilizing the sensors being evaluated. Table 3 showed the RSD% results for repeated determinations.

The linearity of an electroanalytical procedure is measured by how closely the calibration chart of the electroanalytical response to concentration approximates a straight line. As previously stated, the standard calibration graph was generated by measuring five distinct amounts of Ni(II) as standard solutions. It was discovered that the electrode potential [mV] and the logarithm of Ni (II) concentration were linearly correlated. Table 1 includes regression data, correlation coefficients (R^2^), and other statistical metrics.

The quantification limit (LOQ) is calculated by determining the minimum concentration, which could be detected in accordance with the recommendations of IUPAC^48^ and ICH(International Council for Harmonization) Q2 (R1)^49^; below this value, the calibration function was nonlinear. 16.65 × 10^−8^ mol/L was the lowest concentration the nominated electrode can detect. LOD is the minimum amount of the evaluated sample. It was well visible from the LOD results presented in Table 5 that the investigated electrode was extremely sensitive and displayed viable tools for determining small Ni(II) concentrations. LOD was found to be 5.0 × 10^–8^ mol L^-1^, as shown in Table 5, which indicated the high sensitivity of that electrode, which is more than that obtained where the LODs = 2.1 × 10^–7^ mol L^−1^^50^, respectively, and more sensitive than the concentration of 30 μg L^−1^ for LOD^38^.

**Table 5:** Summary of factors that affect the behavior of the proposed electrode.

Parameters	Value
The best content, mg	10
Slope, mVdecade^-1^	29.22 ± 0.12
Plasticizer	TOCP
Response time /Sec	8
Isothermal coefficient mV/C	0.957
pH range	2.0–7.0
lifetime /weeks	9
LOD, mol L^-1^	5.0 × 10^−8^
LOQ, mol L^-1^	16.65 × 10^−8^
Linearity, mol L^-1^	5.0 × 10^−8^–1.0 × 10^−2^

### Comparison of the proposed Ni(II)‑CPE sensor and some of the previous reports

Previously manufactured Ni(II)-selective electrodes and the spinel-based electrode are reported in Table 6, including the pH range, response time, slope, and dynamic linear range. Among the compared sensors, the modified CPE sensor displayed the fastest response (8 s) and long durability (9 weeks) with stable performance within the extended range of pH value (2.0–7.0).

**Table 6:** Comparison between the proposed method of the modified CPE and other studies.

No	Ionophore name	LOD mol L^-1^	Concentration range. (mol L^-1^)	Slope mV decade^-1^	pH range	Lifetime (weeks)	Response Time (s)	Ref
1	Ni^2+-^imprinted polymer (dichloroquinoline-8-ol)	2.0 × 10^–7^	1.0 × 10^–4^-3.0 × 10^–7^	27.90	5.0–7.0	NM	20	^50^
2	5,7,12,14-Tetramethyldibenzo tetraazaannulene (Me4Bzo2TAA)	NM	7.9–10^−6^–1.0–10^−1^	30.00	2.7–7.6	NM	15	^51^
3	1-acenaphthoquinone-1-thio semicarbazone-PVC matrix	5.0 × 10^–7^	1.0 × 10^–6^-1.0 × 10^–1^	29.50	5.0–8.0	12	10	^34^
4	(2E,3E)-2H-1,4-benzothiazine-2, 3(4H)-dione dioxime	1.6 × 10^–6^	1.0 × 10^–6^-1.0 × 10^–1^	29.30	2.0–6.5	NM	10	^52^
5	N,N'-bis-(4-dimethylamino-benzy lidene)-benzene-1,2-diamine	8.0 × 10^–8^	2.0 × 10^−7^–1.0 × 10^−2^	30.00	4.5–9.0	NM	10	^53^
6	Ph_4_Bzo_2_(12)tetraeneN_4_	2.9 × 10^−6^	3.9 × 10^−6^–1.0 × 10^−1^	29.5 0	2.5–7.7	NM	8	^54^
7	3-Hydroxy-N-{2-[(3-hydroxy-N-phenylbutyrimidoyl)-amino]-phenyl}f-N- phenylbutyr-amidine	1.0 × 10^–7^	1.6 × 10^−7^–1.0 × 10^−2^	30.00	2.5–9.5	NM	10	^55^
8	chelex-100 resin	NM	1.0 × 10^−4^–1.0 × 10^−1^	NM	3.5–6.0	NM	15	^56^
9	Electroneutral ion-carriers-barium (ii) and nickel (ii)	5.0 × 10^−5^	5.0 × 10^−5^–1.0 × 10^−1^	NM	3.5–8.0	NM	40	^57^
10	PVC-membrane of macrocyclic N atom	1.8 × 10^−6^	1.8 × 10^−6^–2.3 × 10^−1^	25.00	3.0–7.0	8	10	^58^
11	1-hydroxy-2-naphthadoxime-formaldehyde polymer	NM	4.0 × 10^−5^–1.0 × 10^−1^	NM	3.0–7.5	8	10	^59^
12	Zn-MOF	5.0 × 10^−8^	5.0 × 10^−8^–1.0 × 10^−2^	28.75	3.0–6.5	8	11	^14^
13	Magnesium aluminum spinel -Nps	5.0 × 10^−8^	5.0 × 10^−8^–1.0 × 10^−2^	29.22	2.0–7.0	9	8	This work

*NM: not mentioned.

### Experimental

The chemicals, reagents, and instruments utilized were discussed in the supplementary materials.

### Synthesis of nano spinel (MgAl_2_O_4_)

A modified co-precipitation technique was employed for obtaining nano spinel (MgAl_2_O_4_), as reported in a previous publication^25^. To summarize, suitable distilled water was used to dissolve stoichiometric amounts of magnesium chloride hexahydrate and aluminum chloride hexahydrate. The mixture was then thoroughly combined while being continuously stirred. Ammonia solution was added drop by drop to get the blended solution to a pH of 10.5. The precipitate was repeatedly cleaned using an ethanol and distilled water solution. After that, the precipitates were dried for 24 h at 90 °C in an oven and then calcined for an hour at 1000 °C using a 5 °C/min heating rate. Before being employed as an ionophore in a carbon paste electrode to detect nickel ions in actual samples, the synthesized nano-spinel was subjected to various analytical techniques, including XRD SEM in conjunction with EDAX, TEM, and BET analysis.

### Sensor's modification

They were prepared by mixing graphite powder, solvent mediator, and spinel nanoparticle by 67.3:30.5:2.7 (wt.%). Then, they were put in a dry and cold place until utilization. The surface of the paste was polished using fine tissue to refresh the surface. The obtained response obeys the Nernstian equation: ΔE = (2.030 RT/nF) log [Ni(II)], where ΔE is the difference of potential between measured and constant potential, gas constant (R) is (8.314 J/K.mol), T is the temperature (in K) = 289 K, n is the charge of the ion and F is Faraday constant (96,500 coulombs/mol).

### Samples preparation

#### Cereal breakfast

Dry-aching was carried out for 0.5 g sample; it decomposed at 500 °C for 60 min, followed by a few drops of 65% (v/v) concentrated HNO_3_ or 30% (v/v) hydrogen peroxide H_2_O_2_ then heating for 45 min, as recommended by ASTM international^38^.

### Coca sample

A mixture of HNO_3_ and H_2_O_2_ (3:1% v/v) was added to one g sample. The temperature of the vessels was increased to 200 °C until digestion was completed. The resulting solutions were diluted with 50 mL of deionized water^60^.

### Candy sample

It was digested with microwave-assisted acid digestion. 2.0 g sample is added to the mix of HNO_3_ and H_2_O_2_ (9:1%v/v). The temperature of the microwave instrument increased from room temperature to 200 °C. After digesting, the solution was gently heated, and then the residue was diluted by 0.5 mol l^-1^ HNO_3_^61^.

### Cocaine sample

It was dissolved in deionized water and heated to aid complete dissolution. In the volumetric flask, 2 ml of 2.0% (v/v) HNO_3_ was added to stabilize the solution. Afterward, it was diluted to 30 ml with water^62^.

### Fizzy drink sample

Nitric acid (5M) was added to digest the soft drink sample (1:3%v/v). The added nitric acid removed organic material by decomposing it into carbon dioxide (CO_2_) and converted the metal present into soluble forms according to the equation:

(CH_2_)_n_ + 2HNO_3_ → nCO_2_ + 2NO + 2H_2_O. The mixture was evaporated on a hot plate until digestion. 50 ml of distilled water was added, concentrated by evaporation on a hot plate to half its volume. Subsequently, 25 ml of deionized water was added to make up to 50 ml^63^.

### Chocolate sample

The sample was crumbled and prepared by wet digestion, where a weighed mass of around 2.0 g was heated with concentrated nitric acid for 30 min at temperatures 70–80 °C. The solution was filtered, then the clear solution was transferred in a volumetric flask (50 mL) and diluted to mark with distilled water^64^.

### Candy packages sample

The candy package was digested using the dry ash method (500 °C, six h). About 0.2 g sample was in a ceramic crucible and diluted to 10–100 ml with 0.5 mol l^-1^ HNO_3_ after digesting^61^.

## Conclusion

Nickel is a hazardous metal that may lead to several health problems when consumed in large quantities. Therefore, it is crucial to develop novel techniques for detecting small concentrations of Ni(II) ions. So, in this work, a magnesium aluminum spinel nanoparticle based on carbon paste electrode was used to detect Ni(II) ions in actual samples. The prepared magnesium aluminum spinel nanoparticle using the co-precipitation method, fully characterized by different characterization techniques to confirm the phase composition, nano size, high surface area, and mesoporous characters. The proposed potentiometric sensor revealed excellent response characteristics regarding stability and reversibility. It is clear that the dynamic ranges from 5.0 × 10^−8^ to 1.0 × 10^−2^ mol L^-1^ at room temperature with a detection limit of 5.0 × 10^−8^ mol L^-1^. In addition to a fast response time of 8 s, it can be used for nine weeks with good reproducibility. Also, it is selective against several interference substances. The proposed sensor was successfully applied to determine nickel ions in some samples, including cocaine, candy, coca, chocolate, and fizzy drinks. It also provides a good agreement (accuracy and precision) with the validation approaches, and the results outperform the IUPAC and ICH Q2(R1) recommendations.

### Supplementary Information

Supplementary Information.

## Data Availability

All data generated or analyzed during this study are included in this published article [and its supplementary information files].
