# Supplementary material for: Electrochemical and statistical study of Nickel ion assessment in daily children intake samples relying on magnesium aluminate spinel nanoparticles
Source: Sci Rep. 2024 Jul 16;14:16424. doi: 10.1038/s41598-024-64052-1 (PMC11252383; doi:10.1038/s41598-024-64052-1)
Supplement: Supplementary file 1 — Supplementary Information. [file 41598_2024_64052_MOESM1_ESM.docx]

**Electrochemical and statistical study of Nickel ion assessment in daily children intake samples relying on magnesium aluminate spinel nanoparticles**

## *Maysa R. Mostafa^1^, Gehad G. Mohamed^,1,2^, Omar A. Fouad ^1*^*

1. Chemistry Department, Faculty of Science, Cairo University, 12613, Giza, Egypt.
2. Nanoscience Department, Basic and Applied Sciences Institute, Egypt-Japan University of Science and Technology, New Borg El Arab, Alexandria, 21934, Egypt

**Omar A. Fouad*^1*^*: The** **corresponding author**

E-mail: [oahmed@sci.cu.edu.eg](mailto:oahmed@sci.cu.edu.eg)

**Apparatus**

Using the Quanta FEG250 SEM at the National Research Center in Egypt, a modified carbon paste electrode and a scanning electron microscopy (SEM) image of spinel were reported. The Egypt Nanotechnology Center (EGNC) used the Bruker D8 Discover (Bruker AXS Inc., 35 KV, 30 mA) X-ray diffractometer to record the X-ray diffraction (XRD) using Cu Kα radiation (λ = 1.5406 Å) for two hours with 2θ changing from five to fifty. The speed scan was 0.016 and the step size was 0.02. To find the BET surface area and pore size distribution, gas adsorption tests have been carried out at 77 K using N_2_ as the adsorptive gas.

The materials underwent a four-to-twelve-hour high vacuum evacuation prior to the adsorption trials. Based on the Brunauer-Emmett-Teller (BET) theory, the computation was done and the analysis was carried out using a Nova Touch LX2 analyzer.The inductive coupled plasma mass spectrometry (ICPMS) equipment from Teledyne Leeman Laboratories (Prodigy 7) has been utilized at Cairo University. To evaluate the pH, the Ag/AgCl double-junction reference electrode (HANNA, HI 5311) included with the Model (Hanna, model 8417) was utilized. All glassware and polypropylene tubes with a conical bottom were cleaned with deionized water and then dried.

**Reagents and Chemicals**

All of the study's reagents were analytical reagent grade or higher in purity, depending on the availability. In order to create nano spinel (MgAl_2_O_4_), the following materials were used: ammonia solution (NH_4_OH, Riedel-deHaen, Germany), magnesium chloride hexahydrate (purity 99%, Sigma-Aldrich Chemie GmbH), and aluminum chloride hexahydrate (purity 98%, Sigma-Aldrich Chr. By dissolving the appropriate amount of NiCl_2_.6H_2_O, the solutions of Ni (II) were created. From Acros Organics, USA, nickel chloride was obtained. One was provided with 1-nitro-2-(octyloxy) benzene (o-NPOE). Fluka, on the other hand, received supplies from BDH for hydrochloric acid (HCl), nitric acid (HNO_3_), sodium hydroxide (NaOH), diethylhexyl phthalate (DEHP), dibutyl benzene-1,2-dicarboxylate (DBP), and bis(2-methylpropyl) benzene-1,2-dicarboxylate (DIBP). From Aldrich, graphite powder and tritolyl phosphate (TOCP) were obtained. The following are obtained from El-Nasr Company (Cairo, Egypt): lactose, fructose, maltose, sucrose, starch, ascorbic acid, p-phenylene diamine (PPD), PbNO_3_, AgNO_3_, CoCl_2_.6H_2_O, CdCl_2_, CuCl_2_.2H_2_O, and other metal chlorides including K, Fe, and Cr.


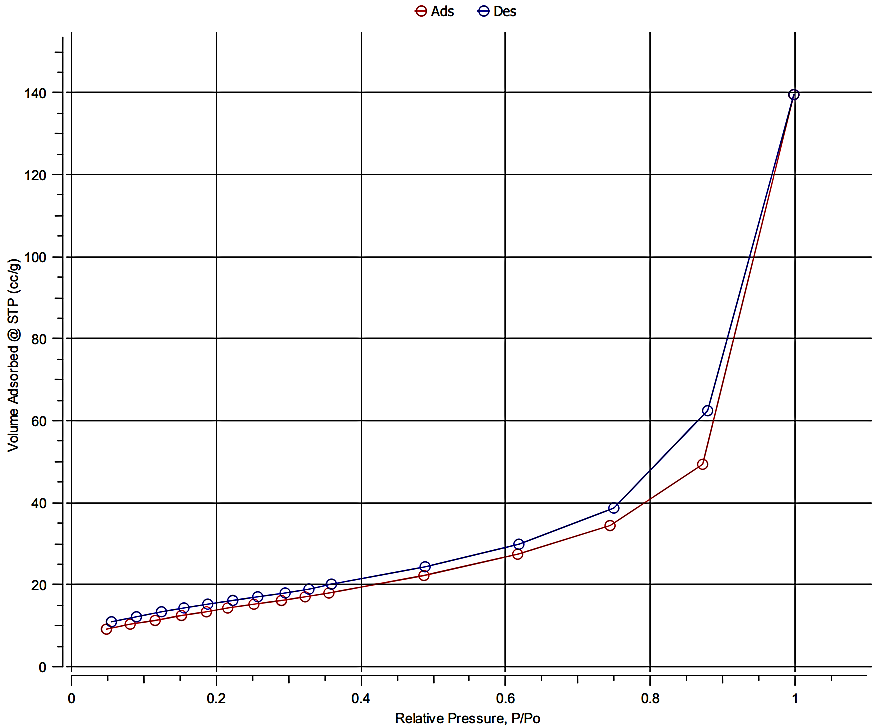


**Supplementary Figure S1.** N_2_ adsorption–desorption isotherms of nano spinel (MgAl_2_O_4_).
